# ‘Unprepared for the depth of my feelings’ - Capturing grief in older people through research poetry

**DOI:** 10.1093/ageing/afac030

**Published:** 2022-03-12

**Authors:** Katrin Gerber, Bianca Brijnath, Kayla Lock, Christina Bryant, Danny Hills, Larissa Hjorth

**Affiliations:** Melbourne Ageing Research Collaboration, National Ageing Research Institute, Melbourne, Australia; Melbourne School of Psychological Sciences, University of Melbourne, Melbourne, Australia; Melbourne Ageing Research Collaboration, National Ageing Research Institute, Melbourne, Australia; School of Allied Health, Curtin University, Perth, Australia; School of Social Sciences, University of Western Australia, Perth, Australia; Melbourne Ageing Research Collaboration, National Ageing Research Institute, Melbourne, Australia; Melbourne School of Psychological Sciences, University of Melbourne, Melbourne, Australia; School of Health, Federation University, Ballarat, Australia; School of Media and Communication, RMIT University, Melbourne, Australia

**Keywords:** grief, bereavement, older people, poetry, mental health, qualitative

## Abstract

**Background:**

Older people are more likely to experience bereavements than any other age group. However, in healthcare and society, their grief experiences and support needs receive limited attention. Through innovative, arts-based research poetry, this study aimed to capture older people’s bereavement stories and the effects of grief on their physical and mental health.

**Method:**

Semi-structured in-depth interviews with 18 bereaved older adults were analysed using thematic and poetic narrative analysis, following a five-step approach of immersion, creation, critical reflection, ethics and engagement.

**Results:**

Research poems were used to illustrate three themes of bereavement experiences among older adults: feeling unprepared, accumulation of losses and ripple effects of grief. While half of participants reported that the death of their family member was expected, many felt unprepared despite having experienced multiple bereavements throughout their life. Instead, the accumulation of losses had a compounding effect on their health and well-being. While these ripple effects of grief focussed on emotional and mental health consequences, many also reported physical health effects like the onset of a new condition or the worsening of an existing one. In its most extreme form, grief was connected with a perceived increased mortality risk.

**Conclusions:**

By using poetry to draw attention to the intense and often long-lasting effects of grief on older people’s health and well-being, this article offers emotional, engaging and immersive insights into their unique bereavement experiences and thereby challenges the notion that grief has an expiry date.

## Key Points

- Cumulative bereavement experiences have compounding and long-lasting effects on older people’s physical and mental health.
- Bereavement challenges can be intensified by pre-existing medical conditions, isolation and lack of social support.
- This is the first study to use research poetry to offer immersive insights into the bereavement experiences of older people.
- Research poetry creates an emotional connection with the audience and thereby encourages open conversations about grief.
- Support services must be tailored to address the unique bereavement needs of older adults.

## Background

As people age, they become more likely to encounter the death of a significant person in their lives and are therefore increasingly likely to experience grief [1]. An American longitudinal study reported that 71% of adults aged over 65 years had experienced at least one bereavement in the last 2.5 years [2]. Census data further showed that, by the age of 75, 58% of women and 28% of men will have been widowed at least once [3]. These numbers will further increase as a result of the coronavirus disease 2019 (COVID-19) pandemic and the ageing population. Grief is therefore an extremely common experience for older adults [1–3].

Independent of age, the stress of bereavement can affect physical and mental health [1, 4, 5], with the potential of compromising people’s functioning for years. For example, it can heighten an individual’s vulnerability to depression [6–8] and post-traumatic stress [7, 9], and negatively affect immune function [10, 11], inflammatory markers [6], neuroendocrine systems [12], weight [4, 13] and sleep [12, 14]. Early work from Thompson et al. [15] showed that 68% of older bereaved adults reported worsened health 2 months after losing their spouse, which was more than double the rate compared with an age-matched control group. Chen et al. [16] added that high levels of traumatic grief 6 months after a death predicted physical health events (e.g. cancer or heart attack) 19 months later. Similarly, high levels of anxiety predicted suicidal ideation after 2 years of bereavement [16].

These physiological and psychological effects of grief may be of particular concern for older people with pre-existing conditions. Yet, when it comes to grief support, older people are often overlooked as the assumption exists that they are well prepared to deal with grief simply because they have encountered so many bereavements throughout their lives [17]. Older people’s unique experiences with bereavement and grief have received little attention. As part of a larger mixed-methods study, this article aims to address this gap by capturing older adults’ bereavement experiences and the effects of grief on their health and well-being by using innovative arts-based methods. Going beyond mere qualitative descriptions, we will allow the reader an immersive glimpse into the emotional world of a bereaved older person through research poetry.

## Method

To gain an in-depth understanding of how older people’s bereavement experiences affected their health and well-being, we interviewed bereaved older adults across Australia. To offer more immersive insights into these bereavement experiences, we developed research poems based on interview transcripts as a creative form of data presentation and research translation [18–22]. This approach is based on established disciplines such as ethnography that have a strong tradition in using creative and multisensory methods to engage participants and the public in innovative ways [23].

### Ethics

This study was approved by the Human Research Ethics Committee of Deakin University (ID: 2019-443, Date: 07.02.20) and received research governance approval from the National Ageing Research Institute (ID: M17, Date: 17.02.20).

### Eligibility

We recruited people aged 65 years or older who lived in Australia, were proficient in English, were able to provide informed consent and had experienced the death of a significant person in their life (e.g. a family member or friend) at least 6 months ago. This time criterion was chosen to protect people in the acute stages of grief. People who lived outside of Australia, required an interpreter or had a cognitive impairment that would prevent them from giving informed consent were excluded during the initial contact.

### Sampling

Non-proportional, purposive sampling was used to recruit participants across Australia. As the majority of interested older people were women, we tried to create a gender balance in the sample by specifically recruiting bereaved older men. Overall, 18 bereaved older adults were interviewed. Of these, 15 older people agreed to have their transcripts used for research poems.

### Recruitment

Participants were recruited through study flyers in public forums such as community notice boards, libraries, councils, general practices, the website of the National Ageing Research Institute, newsletters of the Melbourne Ageing Research Collaboration, Primary Health Networks and social media platforms such as Facebook, LinkedIn and Twitter. Interview participants were offered a gift voucher to thank them for their time.

### Data collection

Interviews followed a semi-structured guide of open-ended questions (see Supplementary File 1), which was developed in collaboration with a multi-disciplinary research team and a consumer representative. After collecting demographic information, interviews with older people began by asking them about their most significant bereavement, how they had coped, how it had affected them physically and psychologically and to what extent they had sought help. Two of the authors (KG and LH) conducted the interviews between May and July 2020, including 13 via Zoom and five via telephone, depending on participants’ preferences. Face-to-face interviews were not possible due to the geographical dispersion of participants across Australia and the second wave of the COVID-19 pandemic in Australia during this time, which saw interstate border closures and strict lockdowns imposed in Melbourne where the researchers were based. Interviews were audio-recorded and varied in length between 31 and 107 min (mean = 60 min). Data collection continued until the topics identified had been explored in sufficient depth and no new themes were raised. Participants were offered their interview transcript to review and all received a resource sheet with contact details of freely available bereavement services should they or someone they know require additional support.

### Data analysis

Interviews were audio-recorded, professionally transcribed and de-identified. Data were analysed using a thematic and poetic narrative approach. Thematic analysis was a continuous, iterative and reflexive process using QSR International’s NVivo. Following the analytical process outlined by Braun and Clarke [24], two of the authors (KG and BB) developed the coding manual by independently coding two interviews each over three consecutive rounds and discussing emerging codes. The refined code manual was then applied by KG and KL to the remaining transcripts. Coded themes were continuously scrutinised and corroborated throughout this process.

**Table 1:** Example of creating a poem from an interview transcript

**Excerpt from an interview transcript**	**Poem**
I could have done more and maybe brought someone in to help. . . I’ve always tried to deal with it myself, and the lack of sleep is the big problem and it has been. I’ve always had it, insomnia, most of my life, but it definitely did get yeah, that’s the one that got worse afterwards [after he died], because you keep busy during the day doing things, you get into bed, and then everything comes. And, you think oh, I should have done this for [husband’s name], I should have done that; you’ve sort of got guilt feelings too. . . And I think well, some of the things when [husband’s name]'s dementia started, and I used to feel angry and frustrated too. I wished I hadn’t but then sometimes you just don’t know what to do. But those sort of things come back when you get to lie in bed and you think.	**Insomnia** Once you start thinking, You can’t sleep. You keep busy During the day. You get into bed, Then everything comes. Guilt feelings I should have done this. I could have done more. Those things come back When you lie In bed And think. (*Susan, 83 years, bereaved wife*)

In addition, poetic narrative analysis was used to illustrate identified themes and connect with a wider audience. Research poetry is a form of arts-based research where poems and poetic statements are developed from existing transcripts [18, 21, 22]. Poems as a creative and non-traditional research output have the potential to make the reader feel the impact of the experience that is being described. This process was led by KG and reviewed by the rest of the research team. We followed the five-step process proposed by Miller [18], who developed them in the context of capturing the experiences of older adults living in residential aged care. The steps included immersion, creation, critical reflection, ethics and engagement. Similar to the initial data reduction phase in any thematic analysis, the first step was for the researcher to immerse themselves in the data by reading and re-reading the transcript in search of keywords and phrases. The second step was to arrange and rearrange these key phrases to craft a poem using participants’ exact words. This process aimed, on the one hand, to illustrate the identified interview themes in an engaging manner, while on the other hand also capturing participants’ unique voice, rhythm, syntax and emphasis. As shown in Table 1, unnecessary phrases and fillers were removed, the sentence and word order were adjusted, while always remaining true to each participant’s experience and way of speaking. Poems were refined through poetic techniques such as rhythm, repetition, metaphors, imagery, synthesis, alliteration and tone [18]. The third step involved critically reflecting on the quality and accuracy of the poem by carefully considering titles, word choice, punctuation, sound and emotion. Step four focused on ethical considerations such as participant engagement, member checking, control and ownership. Participants provided written consent to have their transcripts developed into research poems. They were sent their poems, invited to suggest changes and approve the final versions. They could also decide whether their first name was presented with their poem or a pseudonym assigned. This process was important to ensure that their experiences had been accurately represented. Miller [18] noted: ‘*Research poets must balance poetic impulse with the ethical imperative to remain faithful to the spirit and voice of the transcript, thus ensuring that the transcript poem both functions as a poem and as testimony*’ (p.30). Participants’ feedback was overwhelmingly positive with many feeling that the poems had encapsulated the essence of their grief experiences, describing them as ‘*thoughtful*’, ‘*enchanting*’, ‘*insightful*’ and ‘*magnificent*’. The final step involved engagement with diverse audiences by sharing the poems at conferences, exhibitions, events and online. By way of example, we asked local artists to illustrate some of the poems and created videos of participants and volunteers reading the poetic pieces to bring their bereavement experiences to life [25].

## Results

In-depth interviews were conducted with 11 women and seven men who were on average 74 years old (range: 66–87). Overall, 12 of the 18 interviewed older people reported the death of their partner as the most significant bereavement, three spoke about the death of their parents and three of the death of their child. The most common cause of death was cancer, while others included dementia, accidents, organ failure and suicide. The loss had occurred on average 8 years ago (range 2–27 years).

Three central themes were identified: feeling unprepared, accumulation of losses and ripple effects of grief. While just over half of older adults reported that the death of their family members was somewhat expected, almost all of them felt unprepared for the experience. Especially the timing of the bereavement was unexpected and witnessing the decline and death of their loved one left an element of shock, even years later. One older person described this as being *‘unprepared for the depth of my feelings… never been through anything like this before’ (Richard, 72 years, bereaved partner).* These experiences of witnessing a death are captured in the poems ‘The last breath’ and ‘I couldn’t leave her’.

**The last breath**

We were watching him

Breathing,

The rattle,

Talking to him,

So tired,

Waiting

For the next intake.

He’s not breathing.

. . .

. .

.

I’m a widow.

(Carmen, 71 years, bereaved wife)

**I couldn’t leave her**

I went on the day she died.

She was dead in the bed.

I asked her husband permission,

Could I sit with her?

I couldn’t leave her

I had to sit there

With her

For hours

Before her body was collected.

That was the hardest.

I couldn’t leave her

I kissed her.

She was cold.

Then she was taken away

In the back of the van.

I collapsed,

My sons held me.

I couldn’t leave her.

(Elly, 76 years, bereaved mother)

All older people had experienced multiple bereavements and this accumulation of losses through a cascade of deaths had a compounding effect on their physical and mental health. There were ripple effects of grief associated with the bereavement, e.g. the loss of their role as a partner, parent or caregiver, the loss of identity and sense of family and community. Relatives and friends provided support for the first few weeks, usually until the time of the funeral, after which their lives continued, whereas that of the bereaved person had changed forever. This experience is illustrated in the poems ‘A life to go back to’ and ‘On your own’.

**A life to go back to**

After 6 weeks

Everybody goes away.

They’re attentive

For a while.

‘Are you over it yet?’

Everything that’s normal

Fades.

Then your friends go

Back to their lives.

You’d like to go back

To yours,

But somehow,

It’s not there

For you to go back to.

(Peter, 79 years, bereaved husband)

**On your own**

I had good support

From family and friends,

Before the funeral,

Getting over

All that stuff.

But then,

Of course,

All these people

Drift off

And go home

And you’re left

On your own.

(Richard, 72 years, bereaved partner)

**Figure 1:**
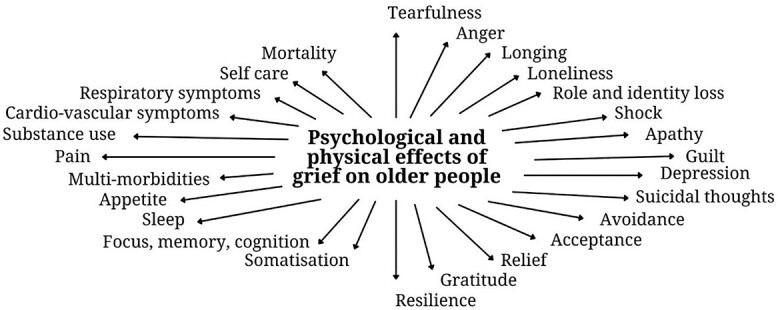
Overview of identified psychological and physical effects of grief on older people.

The ripple effects of grief had considerable and sometimes long-lasting consequences for older people’s health. One participant said: *‘There are impacts beyond the death that resonate down the years’ (Helen, 68 years, bereaved mother).* As summarised in Figure 1, most descriptions focussed on emotional and psychological consequences of grief and ranged from challenging feelings like anger, apathy and guilt to positive emotions like acceptance, gratitude and relief. Many participants also reported physical health effects like the onset of a new condition or the worsening of an existing one, such as difficulty focussing, changes in appetite and sleep, breathing problems, heart racing and physical pain. These responses were particularly noticeable for people with pre-existing medical conditions. Lack of self-care and personal hygiene was often noticed by primary care staff as a physical consequence of grief. Substance use was also discussed, especially the use of alcohol and sleeping pills to self-medicate. In its most extreme form, grief was connected with a perceived increased risk of mortality. This ranged from suicidal thoughts to apathy and indifference about life, as shown in the poem ‘Stand back’. For many, the challenge was to find new meaning in life while being confronted by the void left by the loss. This is illustrated in the poem ‘The empty chair’.

**Stand back**

I found myself standing quite a way back

On the train platform

When the train came in,

Just in case

I was tempted

To jump.

I don’t think they were suicidal thoughts.

But I wouldn’t have done much

If death was imminent.

If the truck was heading towards me,

I might not get out of the way.

But I wasn’t going to jump

In front of it.

(Richard, 72 years, bereaved partner)

**The empty chair**

My friend rang me

Every day

After he died

Wanted to know

I was out of bed

Made me talk

Kept me alive

It was painful

To be in the house

The long evenings on your own

Everywhere was a reminder

He wasn’t there

You could still see him

Hear him

His chair

Where he sat

Empty

I wanted to get out

Escape the feeling

Of being punched in the gut

I still wanted to live

Stay alive for the kids

These children,

They need me

Stay alive

Live

There is only one problem

I don’t know what to do

With the rest of my life

(Jenny, 66 years, bereaved wife)

Many participants felt that experiencing a significant death could influence a person’s will to live. Some had encountered couples who died in short succession of one another. For example, one participant described how his father died on a Monday, after which his mother suddenly deteriorated and died the following Saturday. This increased mortality risk after a significant bereavement is often described as the ‘widowhood effect’ [26–28] or ‘dying of a broken heart’ [4, 29].

## Discussion

To our knowledge, this is the first study to use poetic narrative analysis and research poetry to offer emotional insights into the bereavement experiences of older people, while also capturing the effects on their physical and mental health. Having their personal stories developed into poetry helped participants to feel heard and noticed in their grief. The rise of non-traditional and arts-based methods has taken on much gravity with the growing significance of public engagement and impact agendas. Arts-based methods can help to probe visceral feelings and experiences in ways that are creative and immersive [23]. Creative techniques such as poetry can also be used to elicit participants’ responses to discuss difficult topics including death, grief and loss.

We found that the number of deaths older adults had encountered over their lifetime accelerated with increasing age. This is not surprising but, contrary to the expectations of society and healthcare staff [17], this accumulation of losses did not make older people more resilient or prepared for the next loss. Instead, the experienced cascade of bereavements had a compounding effect on their physical and mental health. While participants’ most significant loss occurred on average 8 years ago, some still struggled to redefine their identity and find new meaning in life. This highlights the importance of continuous, long-term psychosocial mental health and bereavement support for older people after a significant death to prevent or at the very least reduce the potential ripple effects of grief.

More attention also needs to be given to the physical manifestations of grief, such as pain, cardio-vascular symptoms and sleeping problems, potentially leading to increased substance use. This is a particular concern for older age groups where the use of alcohol or sleeping pills can increase the risk of drug interactions and falls [30, 31]. Healthcare staff must consider that physical symptoms may relate to bereavement stress and use holistic approaches of care that support both older people’s physical and mental health.

The increased risk of mortality after a significant bereavement, also known as the ‘widowhood effect’ [26–28], may be attributed to broad biopsychosocial mechanisms that can trigger a worsening of mental, cognitive and functional health [8, 32–34]. It can relate to sudden cardiovascular events, accidents, cancers caused by increased alcohol use and smoking, or acute illness caused by a reduced immune function following a bereavement [8, 26–28, 32–34]. Pre-existing medical conditions, lack of support and social isolation make older people a particularly vulnerable group for such negative outcomes of grief. Furthermore, in this study, suicidal thoughts after a bereavement were predominately discussed in regard to older men who, according to the World Health Organisation, have the highest age-specific rate of suicide [35]. This emphasises the need for targeted interventions tailored to the needs of older bereaved adults.

### Limitations

While this study offered new and immersive insights into the lived experiences with grief from the perspective of older people, it must be acknowledged that the interviews were conducted during the second wave of the COVID-19 pandemic in Australia. This may have impacted participants’ mental health and consequently influenced their responses. Furthermore, a volunteer bias must be considered in that people who did not experience any significant grief effects may have been less likely to volunteer for this study. Also, we must consider that those who are more disposed to poetry may have been more likely to consent to have their transcripts developed into research poems. Although this is less likely given that almost all of the interview participants (15/18) consented to this. However, we do not know whether any participants had a particular affinity towards poetry and how this may have influenced their willingness to be involved in the poetry component of the study. Finally, we note that the process of generating the research poems is a subjective one and as an arts-based method does not seek objectivity either. Instead, the aim is to illustrate the identified effects of grief and create an emotional connection between the older bereaved person and the audience through poetry.

## Conclusions

Understanding the complex experiences of grief requires using alternative and innovative methods. Creative approaches can help elicit feelings and responses often left tacit. Arts-based techniques like poetry can provide a space to develop empathy in others, thus allowing avenues for taboo topics like grief to be discussed more openly. Bereavement affects people of all age groups but older people face specific challenges due to the accumulation of losses which can be intensified by pre-existing medical conditions, isolation and lack of social support. Bereavement support services must therefore be tailored to older people’s unique needs, while acknowledging that every new bereavement experience may re-awaken memories of previous losses and thereby intensify their grief. Furthermore, we must address ageist stereotypes assuming that older adults are well prepared to deal with grief because they have encountered so many deaths. In this regard, research poetry can be an effective way to draw attention to the bereavement needs of older people and challenge the notion that grief has an expiry date. By transforming our participants into poets, we not only empower them through co-creation and storytelling as a way to make sense of the world but we provide nuanced models for understanding one of the most complex experiences in life: grief.

## Supplementary Material

aa-21-1745-File002_afac030
