# Supplementary material for: ‘Unprepared for the depth of my feelings’ - Capturing grief in older people through research poetry
Source: Age Ageing. 2022 Mar 12;51(3):afac030. doi: 10.1093/ageing/afac030 (PMC9171723; doi:10.1093/ageing/afac030)
Supplement: aa-21-1745-File002_afac030 [file aa-21-1745-file002_afac030.docx]

***‘Unprepared for the depth of my feelings’ -
Capturing grief in older people through research poetry***

**Supplementary file 1 – Interview schedule older people**

***Bereavement***

- Could you tell me a little bit about the person who died and how this happened?
  - Prompts: Type of relationship to the deceased? How long have you known [name]? When did they die? What did they die of? Was their death expected or unexpected for you?

***Coping and mental health post-bereavement***

- How did you cope after [name] had died?
- How did their death affect your health, including your mental and physical health?

***Healthcare use and awareness of available support services:***

- Have you used any healthcare and bereavement support to cope with the death of [name]?
  - If yes, which ones? How often? What did you find helpful? What was not helpful?
  - If not, what were the reasons for not seeking bereavement support?
- Have you spoken to your GP/ nursing staff about how you felt after [name] had died?
  - If yes, how did you describe how you were feeling? How did they respond?
  - If not, why not?
